# Wormley’s Micro Chemistry of Poisons

**Published:** 1885-07

**Authors:** 


**Wormley's Micro Chemistry of Poisons.** Including their Physiological, Pathological and Legal Relations. With an Appendix on the Detection and Microscopic Discrimination of the Blood. Adapted to the Use of the Medical Jurist, Physician and General Chemist. By THEODORE G. WORMLEY, M.D., Ph.D., LL.D., Professor of Chemistry and Toxicology in the Medical Department of the University of Pennsylvania. *A new, revised and enlarged edition.* With new illustrations. Large 8vo, extra, cloth, $7.50. Sheep, $8.50. J. B. Lippincott Company, Philadelphia.

The appearance of a new edition of this valuable work will be welcomed by all interested in the particular field of science to which this book is devoted, and in which it has no equal. It must not be supposed, however, that the scope of the work is confined to the chemical properties of the poisons as revealed by the microscope. It is, in fact, a complete text-book on Toxicology, and well adapted in its arrangements to the use of the physician and jurist. The present edition has been much enlarged and brought fully up to date. We need say nothing of the great beauty and correctness of the steel engravings, for all who have examined them have united in expressions of admiration. Three new steel plates and a very beautiful chromo-lithograph of blood spectra have been added in this edition.

